# Infection Biomarkers Based on Metabolomics

**DOI:** 10.3390/metabo12020092

**Published:** 2022-01-19

**Authors:** Rúben Araújo, Luís F. N. Bento, Tiago A. H. Fonseca, Cristiana P. Von Rekowski, Bernardo Ribeiro da Cunha, Cecília R. C. Calado

**Affiliations:** 1 ISEL—Instituto Superior de Engenharia de Lisboa, Instituto Politécnico de Lisboa, 1959-007 Lisbon, Portugal; rubenalexandredinisaraujo@gmail.com (R.A.); tiago.alexandre.hf@gmail.com (T.A.H.F.); crisvr66@hotmail.com (C.P.V.R.); bernardo.cunha@tecnico.ulisboa.pt (B.R.d.C.); 2 Medical Urgency Unit, São José Hospital, Centro Hospitalar Universitário de Lisboa Central, 1150-199 Lisbon, Portugal; luis.bento@chlc.min-saude.pt; 3 CHRC, CEDOC, NOVA Medical School, NMS, Universidade Nova de Lisboa, 1169-056 Lisbon, Portugal; 4 CIMOSM—Centro de Investigação em Modelação e Otimização de Sistemas Multifuncionais, ISEL, 1959-007 Lisbon, Portugal

**Keywords:** infection, metabolomics, biomarkers, diagnosis, prognosis

## Abstract

Current infection biomarkers are highly limited since they have low capability to predict infection in the presence of confounding processes such as in non-infectious inflammatory processes, low capability to predict disease outcomes and have limited applications to guide and evaluate therapeutic regimes. Therefore, it is critical to discover and develop new and effective clinical infection biomarkers, especially applicable in patients at risk of developing severe illness and critically ill patients. Ideal biomarkers would effectively help physicians with better patient management, leading to a decrease of severe outcomes, personalize therapies, minimize antibiotics overuse and hospitalization time, and significantly improve patient survival. Metabolomics, by providing a direct insight into the functional metabolic outcome of an organism, presents a highly appealing strategy to discover these biomarkers. The present work reviews the desired main characteristics of infection biomarkers, the main metabolomics strategies to discover these biomarkers and the next steps for developing the area towards effective clinical biomarkers.

## 1. Introduction

The diagnosis of an infection is usually based on clinical, laboratorial and imaging data. To support diagnosis, either a positive culture or a positive molecular diagnosis test may be conducted. However, culture-based diagnosis is time consuming and laborious. Molecular tests, e.g., by polymerase chain reaction (PCR) for a specific agent, are prone to false positives and false negatives. The high sensitivity of these tests may exceed clinical significance leading to false positives, where testing too early, targeting inappropriate specimen type, and suboptimal specimen collection may result in false negatives [1,2]. For example, a multiplex PCR-based study covering 25 bacterial and fungal pathogens over blood samples of 306 participants only identified 69.7% of the cases in relation to the culture-based method (46 vs. 66, respectively, *p* = 0.0004) [3].

Systemic signs, such as fever, tachycardia and leukocytosis, are nonspecific, that is, they are also present in other non-infectious inflammatory conditions, such as trauma, surgery, burns, acute respiratory distress syndrome (ARDS), deep vein thrombosis, pulmonary embolism, and pulmonary infarction, among others. These non-infectious inflammatory conditions may result, for example, in the release of cytokines such as interleukin-1 (IL-1), interleukin-6 (IL-6), tumor necrosis factor alpha (TNF-α), and gamma interferon (INF-γ) [4], which mask those specific to the inflammatory response to infection. For all of these, infection biomarkers have been searched, as are the cases of procalcitonin (PCT) or C-reactive protein (CRP), among others. However, these markers are also not specific to infection, do not discriminate the causative agent, cannot be used to evaluate therapeutic responsiveness, nor accurately predict disease outcome.

For example, recent meta-analysis studies compared the cluster of differentiation 64 (CD64), PCT and IL-6 as biomarkers for sepsis diagnosis in adult patients [5], and to predict gram-negative bloodstream infection [6]. CD64 showed the highest diagnostic value for sepsis, where PCT showed better diagnostic potential for sepsis diagnosis in patients with severe conditions compared with non-severe conditions [5], with an area under the curve (AUC) of a receiver operating characteristic (ROC) curve, for both studies, higher than 0.80. From the comparison between PCT, CRP and IL-6, to predict gram-negative bloodstream infection, PCT showed better predictive performance (AUC = 0.80) [6]. However, PCT cannot reliably differentiate sepsis from systemic inflammatory response syndrome (SIRS) in critically ill adult patients [7], febrile neutropenia and hematological malignancy [6,8], or even to discriminate between ventilator-associated tracheobronchitis and ventilator-associated pneumonia [9]. Furthermore, low PCT can also be present in subacute bacterial endocarditis and in localized infections [10].

The identification of biomarkers to guide antibiotic use is also important. There is an overuse of antibiotics in patients in risk of severe outcomes and in critically ill patients, especially with broad-spectrum antibiotics. The prolonged treatment with unnecessary antibiotics, leads to increased antibiotic-resistance, toxicity, allergic reactions, and drug-drug interaction, all the while resulting in the increase of hospitalization duration. Ideally, a biomarker will predict infection and the type of causative agent, e.g., viral versus bacterial, thereby reducing unnecessary use of antibiotics and, consequently, the metabolic burden of already fragile patients. The Stop Antibiotics on Procalcitonin guidance Study (SAPS) pointed that PCT guidance led to a reduction in the duration of antibiotic treatment, as well as daily doses in critically ill patients with a presumed bacterial infection, and on a significant decrease in mortality [11]. However, multiple other studies performed to assess PCT-guided antibiotics discontinuation and mortality in critically ill patients revealed low-certainty evidence, with a high risk of bias, as showed by the conflicting data in the systematic review and meta-analysis [12,13,14,15,16].

Another reason why a biomarker of therapeutic responsiveness is desirable, is to predict antibiotic resistance while minimizing the use of broad-spectrum antibiotic. In critically ill patients, when there is a suspicion of infection, the current standard of care is to administrate a broad-spectrum antibiotic while waiting for pathogen identification and susceptibility to antibiotics. In cases of infection with a strain that is drug-resistant, the delay from sample collection to drug susceptibility assay allows the infection to progress, which leads to a dysregulation of the immune system [17], thereby exacerbating mortality [18].

Currently, antibiotic resistance is one of the biggest health threats on a global scale. For example, the European Antimicrobial Resistance Surveillance Network pointed out that in the European Economic Area (EEA), more than 670,000 infections per year occur by antibiotic resistant bacteria, directly resulting in 33,000 deaths, whose healthcare related costs were estimated at EUR 1.1 billion [19]. It has been pointed out that in the EEA region, half of the *Escherichia coli* isolates and more than a third of the *Klebsiella pneumoniae* isolates were resistant to at least one antimicrobial group and combined resistance was frequent. Several countries reported resistance to the last line of antibiotics as carbapenem, as above 10% for *K. pneumoniae*.

It is also critical to discover good biomarkers of disease severity and progression, such as prediction of patients going to intensive care unit (ICU), or the need for mechanical ventilation or even predicting the short-term and long-term mortality [20]. The early identification of patients at risk of developing severe disease allows more frequent monitoring of those patients, as well as an early or reinforcement treatment that can improve patient survivability and optimize short and long-term management strategies. The assessment of severity and disease progression can be based on clinical scores, such as with the pneumonia severity index (PSI), and acute physiology and chronic health evaluation (APACHE II), among others. However, these scores present limitations. For example, PSI score, based on 20 demographic, comorbid and clinical variables, enables the patient’s stratification in risk classes based on risk of death within 30 days. Despite PSI validation, this score overestimates severity in older patients with comorbidities and may underestimate severity in young healthy patients with severe respiratory failure as observed in the 2009 influenza A (H1NB1) pandemic [21]. Most of the clinical scores presents limitations to stratify among patients with a high risk of death [21,22,23]. Due to all these limitations, biomarkers have been searched to predict disease severity and progression. Furthermore, biomarkers present inherent advantages over clinical scores as they do not depend on extended clinical data, including laboratorial multi-analysis and, consequently, could be more rapidly and easily obtained.

**Figure 1:**
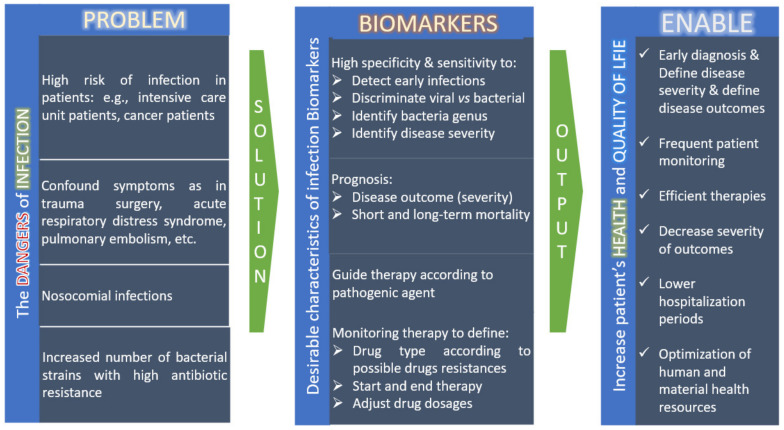
Types of infection Biomarkers according to the clinical problem.

In short, regarding the management of patients with infections, it is relevant to define biomarkers that effectively will help physicians to diagnose the infection, to predict the causative agent, the disease severity and progression and to monitor the antimicrobial therapy (Figure 1). These biomarkers should be especially applicable in patients in risk of developing severe diseases, critically ill patients, and those presenting confound symptoms that may result in under and over diagnosis of infection. More specific and sensitive biomarkers of infection have great potential to decrease severe outcomes, minimizing hospitalization times and significantly improve patient survival (Figure 1).

This section may be divided by subheadings. It should provide a concise and precise description of the experimental results, their interpretation, as well as the experimental conclusions that can be drawn.

## 2. Metabolomics Overview

The limitations associated to current biomarkers result from the wide variability of physiological states among the human population, especially in high-risk and critically ill patients. Methods that enable a holistic search for molecular patterns, particularly in high-dimension biological data of complex inter-related physiological states, have *a priori* advantages that can facilitate the discovery of more efficient biomarkers. Systems biology enables this type of search, as it integrates a wide span of biological knowledge in complex models of regulatory biological networks [24]. From the technical developments of the last two decades, including the analytical techniques and associated bioinformatic tools, the omics sciences enabled the exponential increase of the associated knowledge to biological systems. Omics technologies are routinely used to characterize a defined system, e.g., at the cell level or of a defined biofluid, and at specific conditions, including but not limited to genes (genomics), expressed genes (transcriptomics), proteins (proteomics) and metabolites (metabolomics) [25].

Metabolomics concerns the systematic study of the composition in chemical species of a specific biological system with a molecular weight usually <1500 Da. These molecules act in metabolic pathways associated to normal and pathological processes, as primary, intermediate and/or end products of metabolism, thus retrieving the metabolic phenotyping of a given system [26,27,28]. Therefore, of all the omics sciences, metabolomics is the most functional approach as it provides a direct vision of the functional metabolic outcome of an organism’s activities.

### 2.1. Major Analytical Techniques

The major analytical techniques used to acquire metabolomic data are proton (^1^H) nuclear magnetic resonance spectroscopy (NMR), direct injection (DI) on mass spectrometry (MS) based equipment, to tandem mass spectrometry equipment (MS/MS), or even a separation technique as liquid (LC) or gas chromatography (GC), capillary electrophoresis (CE) associated to MS or to MS/MS. More recent techniques opened the door to imaging metabolomics, which culminates in the localization of defined metabolites within tissue samples [29,30].

The diverse metabolomic techniques present complementary information as some classes of chemical compounds are ‘better’ detected by specific techniques, such as between NMR and chromatographic based technique and even between LC and GC-based ones [23,31]. Therefore, when possible, there are clear advantages in using multiple techniques if the goal is to understand the underlining biological mechanisms or to discover new unrevealed mechanisms. If a defined class of chemical compounds are targeted, then a specific technique can be selected. The specifications of these techniques (e.g., low, and high-resolution MS based techniques), and their intrinsic advantages and limitations are not the focus of this manuscript, and have been reviewed elsewhere [26,29,30,32].

### 2.2. Untargeted versus Targeted Analysis

**Figure 2:**
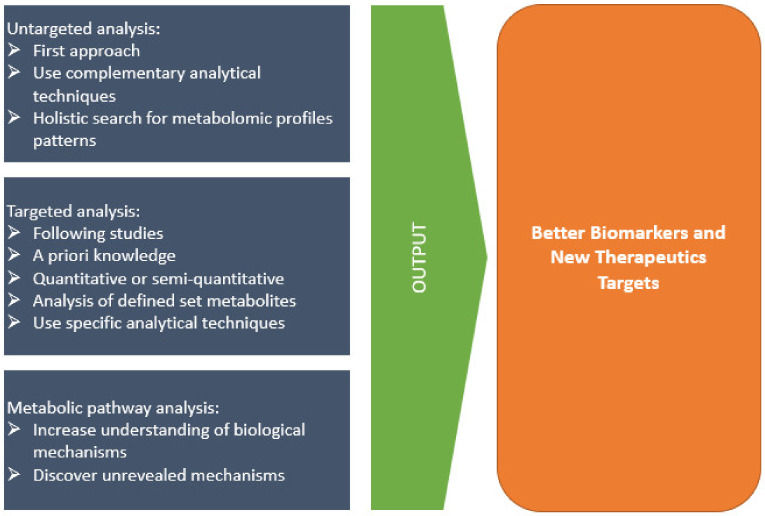
Metabolomics function modes (untargeted, targeted, and metabolic pathway analysis) towards biomarkers discovery.

As a function of *a priori* knowledge of the system, metabolomics may be conducted by untargeted or targeted workflows (Figure 2). An untargeted workflow follows a holistic approach and is driven to retrieve as much information as possible of a given system, e.g., for a given biological sample, the first approach can be conducted by pattern-recognition methods to discover molecular signatures that discriminate pathophysiological states. The main goal is to acquire an overview of potential metabolites present in the system to discover unanticipated perturbations and possible interconnected metabolites, i.e., involved pathways, and even to discover metabolic mechanisms [32]. Depending on the technique used, diverse classes of metabolites can be searched.

Target metabolomics are usually applied to validate *a priori* knowledge. That is, a targeted analysis is usually applied if some previous information points to a specific set of metabolites, where a quantitative (determining absolute concentrations) or semi-quantitative (i.e., analyzing relative intensities) is conducted (Figure 2). The set of target metabolites may be chosen according to diverse criteria, e.g., associated to target metabolic pathways.

### 2.3. Biomarkers

A biomarker, per definition, is a characteristic that can be analyzed, quantified and associated to a defined phenotype [33]. In other words, a biomarker can be a metabolite, or a set of metabolites, or can be a molecular feature, e.g., a spectrum as exemplified, for example, in Cunha et al. [34]. In this last case, the biomarker is not necessarily associated to a defined molecule, and consequently will not be possible to associate it to a metabolic pathway. Despite this, biomarker acceptance by the clinical community increases with the identification of specific biomolecule(s) and its association to a metabolic pathway. The knowledge obtained from the biological mechanisms associated to the metabolic pathways will improve understanding of pathophysiological process that, per se, can lead to the enhancement of prediction outcomes and provide new biomarkers and therapeutic targets. In other words, there are diverse advantages to consider the metabolic information extracted from metabolomics, and even to develop metabolomic studies focusing the understanding of the biological system.

It is suggested to apply metabolomics during the biomarker discovery phase. If biomarkers are a set of molecules, then their detection during the biomarker validation phase or for subsequent clinical practice, can be conducted based on cheaper, faster, and even point-of care devices, as based on biosensors. However, the NMR or MS spectra can be used as biomarkers. In this case, the NMR spectroscopy and GC or LC-MS analysis cannot be replaced. Furthermore, biomarkers of infection, prognosis, and those to guide and monitor therapy, could be different, which suggests the potential need for this type of analytical techniques after the biomarker discovery phase. With that in mind, there have been advances in the miniaturization of NMR equipment towards their application in point-of-care devices [35,36].

## 3. Metabolomics to Discover Biomarkers of Infection

### 3.1. Predict Infection, the Causative Agent, Diseases Severity and Disease Outcome

With the goal of discovering biomarkers based on minimal invasive analysis, the present examples will focus on biofluids analysis. Table 1 points to some works based on biofluids metabolomics. The selected examples highlight diverse types of biomarkers and diseases.

There are diverse studies of metabolomics of plasma, serum, or urine, that enabled to build good models to predict infection in relation to healthy volunteers, and infection among non-infected critically ill patients and patients presenting confound pathophysiological processes (e.g., mechanical ventilated patients, cancer patients with neutropenia, etc.,) (Table 1). For example, Mickiewicz et al. [44], based on serum NMR analysis from a mixed pediatric cohort (including neonates, infants, toddlers and school age till 11 years old) with septic shock (n = 60), SIRS (n = 40) and healthy controls (n = 40), were able to develop models that discriminated septic shock from healthy (AUC = 0.98), SIRS from healthy (AUC = 0.95) and even septic shock from SIRS (n = 0.82).

Concerning predicting diseases outcomes, most of metabolomics of infection predict mortality. Besides the examples pointed in Table 1, it is worth pointing out the CAP and Sepsis Outcome Diagnostics (CAPSOD) study that enrolled 1152 patients including non-infected SIRS and sepsis, from emergency units from three hospitals, from which a total of 781 patients with sepsis and 112 with non-infected SIRS were subsequently eligible [53]. A model, based on five metabolites and age and hematocrit, predicted survival better than the clinical scores based on sequential organ failure assessment score (SOFA) and APACHE II. For example, the accuracy, positive prediction values (PPV) and negative predictive values (NPV), for the validation dataset, based on this set of markers, at hospital admission was 74.5%, 94.1% and 35.3%, respectively. SOFA predicted survivability with an accuracy, PPV and NPV of 61.8%, 75% and 30%, respectively. APACHE II predicted survivability with an accuracy, PPV and NPV of 73.9%, 93.9% and 23.1%, respectively. A second validation work, was conducted using an independent cohort from another institution and based on a different enrolment protocol (RoCI study), leading to an AUC, accuracy, PPV and NPV of 0.734, 74.6%, 83.6% and 55.0%, respectively. In a following study, based on an in vivo model conducted on *Macaca fasticularis* infected with *E. coli*, Langley et al. [54] identified a set of metabolites in the plasma that enabled to discriminate non-infected SIRS from sepsis on the CAPSOD and RoCI cohorts after 24 h of hospitalization with AUCs of 0.821 and 0.786, respectively.

Due to the current relevance of the Coronavirus disease 2019 (COVID-19), it is important to mention metabolomics studies focusing these patients. For example, Robert et al. [50] based on serum LC-MS/MS, with a cohort of 120 patients, and a independent validation set with other 90 patients, develop good models to predict COVID-19 disease severity (AUC = 0.860), and mortality (AUC = 0.830). Delafiouri et al. [52] based on a larger cohort study (with 1165 participants from three Brazilian epicentres), developed good models predicting the infection (specificity >0.96, sensitivity >0.83) and disease severity (specificity >0.80 and sensitivity >0.85).

In short, all the examples given in this section points the potential value of metabolomics in biomarkers discovery. It is also pointed the potential application of these biomarkers for therapy guiding as they could predict infection, the disease severity/state, and the causative agent.

### 3.2. Metabolic Information

The present work main goal is to evaluate how metabolomics can potentiate the discovery of infection biomarkers. Despite this, it is worthy to point out some examples emphasizing how metabolomics could increase knowledge associated to infection and to point potential therapeutic solutions. Bernatchez and McCall [55], reviewed lung metabolomics in bacterial and viral infections. The authors identified common features, for instance in bacterial infections as an increase in oxidized glutathione, most probably due to inflammatory processes, and an increase in amino acids (lactate, glutamate and aspartate) that may reflect increased proteolysis. These authors also pointed out exceptions to these observations, that result from the pathogen specific action. Mechanistic interpretation associated to the differential production of a defined metabolite in lower respiratory tract infections, as in CAP and chronic obstructive pulmonary disease has been reviewed in, e.g., in Nickler et al. [56] and Zurfluh et al. [57].

Bernatchez and McCall [55] also pointed common features in viral infections that included variations on uridine, sphingosine, sphinganine, and kynurenine, adenosine monophosphate and threonine, mannitol, myo-inositol, and glyceric acid. The authors hypothesized that alterations in amino acids, lipids, and nucleosides/nucleotides most probably reflected the host production of new viral particles, whereas the immunomodulation associated to pro-inflammatory (e.g., sphingosine, which is metabolized to sphingosine-1-phosphate) and anti-inflammatory molecules (e.g., kynurenine), contribute directly to the clinical outcome, i.e., the pathogenesis. Patients with ARDS and with H1N1 influenza A pneumonia had decreased serum levels of glucose, alanine, glutamine, methylhistidine and fatty acids concentrations, and elevated serum phenylalanine and methylguanidine concentrations in relation to patients without ARDS [58]. H1N1 pneumonia patients showed increased plasma concentrations of dimethylamine, β-alanine, formate, and quinic acid and a decreased concentration of alanine versus the other two cohorts, one with an bacterial infection and another with non-infected ventilated ICU patients [23]. The best predictive models of mortality were based on lipid molecules in relation to models based on non-lipid molecules, reflecting the relevance of lipid molecules on infection, since diverse lipids are mediators of inflammatory processes. Since lipids are also the main constituent of surfactant in the lungs, lipids variation in plasma could also reflect loss of structures and function of alveolar epithelial cells [59].

The increased knowledge associated to metabolic processes along disease progression, can be used to explore potential therapeutic targets. For example, Wozniak et al. [47], based on serum analysis (n = 200) from patients with bacteremia by *S. aureus*, identified thyroxine (T4) as the most promising feature associated with mortality. Based on this, authors subsequently observed in a mouse model infected with *S. aureus*, the survivability increase after the stimulation of both thyroid and adiponectin signaling pathways.

Diverse metabolomics work focuses COVID-19 patients. For example, Lorente et al. [60], pointed the serum metabolomic specificity of COVID-19 patients with ARDS relative to patients with ARDS due to influenza A pneumonia. Other authors explored the metabolome of COVID-19 patients relative to healthy volunteers, to increase the knowledge associated to the disease. For example, Shen et al. [61], due to the high impact of the infection on downregulating more than 100 lipids, propose drugs inhibiting lipid synthesis as a potential therapeutic regime. Drogan et al. [62], based on significant differences between patients and healthy controls in terms of purine, glutamine, leukotriene D4, and glutathione metabolisms, proposed the use of selective leukotriene D4 receptor antagonists, targeting purinergic signaling as a therapeutic approach and glutamine supplementation to decrease severity. Paez-Franco et al. [63], have also proposed the potential relevance of amino acid supplementation during the infection due to alterations with diverse amino acids along disease progression. Su et al. [64], observed a sharp difference in the plasma metabolome between mild to moderate COVID-19, and a surprising similarity between moderate and severe COVID-19. The shift was marked by the loss of lipids, amino acids, and xenobiotic metabolism along the disease progression from mild to severe. Such phenotypes were observed in moderately ill patients and were only relatively increased in severe patients. This observation, lead authors to propose that therapeutic interventions at the stage of moderate disease are likely to be most effective.

## 4. Metabolomics Integration with Other Omics

For a better understanding of the complex interrelationships between pathophysiological processes, including infections, other inflammatory processes and other systems dysregulation, it is desirable to integrate metabolomics with other omics sciences.

Transcriptomics associated to metabolomics can identify up-stream regulators of metabolic pathways, enabling a deeper understanding of underlying biologic processes [64,65,66]. For example, serum metabolomics and transcriptomics was used to predict sepsis diagnosis and prognosis (e.g., death) [54]. Transcriptomics supported the hypothesis pointed out by metabolomics, namely that mitochondrial dysfunction may lead to problems in β-oxidation and the increase in acyl-carnitines. Example of support by transcriptomics data was e.g., that acyl-phosphatidylcholine, and acyl-diacyl-glycerophosphocholine and carnitine esters had a strong correlation to genes involved in branched-chain amino acid degradation, b-oxidation, and peroxisomal lipid oxidation.

Serum metabolomics and proteomics was conducted to predict the mortality risk by *Staphylococcus aureus* bacteremia [47]. The integration of both omics sciences resulted in the identification of over 10,000 features from 200 serum samples, and importantly provided a comprehensive view of the early host response to infection while enabling prognosis biomarkers that exceed the predictive capabilities of those previously reported. The integration of metabolomics and proteomics was also conducted to screen a biomarker of CAP severity, over 240 serum and plasma samples within a multicenter clinical study focusing hospitalized patients. Omics data were associated with CAP patients stratified according to the SOFA score, and adjusted for age, BMI, sex, smoking and technical variables. Both proteome and metabolome profiles revealed strong predictabilities of CAP severity. The best prediction models involved the lipid metabolism and metabolites associated to dysfunctions of respiratory, renal, coagulation and cardiovascular systems [67].

Jefferies et al. [68] designed a clinical trial that integrated omics sciences with the aim of discovering biomarkers of severe acute respiratory tract infection, in infants, due to respiratory syncytial virus and respiratory sequelae [69]. The clinical trial includes an analysis of diverse types of biological samples (nasopharyngeal, blood, buccal, stool, and urine), by genomics, host immune response, transcriptomic, proteomic, metabolomic and epigenomic.

## 5. Conclusions

Metabolomics presents a highly appealing strategy to discover infection biomarkers, i.e., to develop models to predict the infection, the causative agent, disease severity and disease outcome, and consequently also to guide and monitor antimicrobial therapy. Due to the critical need to discover infection biomarkers, especially for patients at risk of developing severe diseases, there are some very interesting studies focusing on: the prediction of infection in early stages, as in patients in ICUs; predicting infection among confounding clinical outcomes such as non-infectious inflammatory processes; to discriminate the causative agent; and to predict disease severity and disease outcome, such mortality. However, most of these studies are of small dimension, do not use independent data sets for validation, and are not multicenter. Furthermore, in general, these studies include few sub-populations with individuals in each group with homogenous phenotypes, that is, most of the studies do not embrace confounding factors, nor consider the potential effect of factors such as diet, ethnicity, medication, among others. Therefore, most of the studies point more to the metabolomics potential rather than to a clinically applicable biomarker. It is, therefore, critical for better study designs, including higher diversity of pathophysiological states as comorbidities, with larger dimensions, and multicenter. To maximize the metabolomic potential, it is also relevant to develop metabolic pathways analysis and integrate this knowledge with other omics sciences, such as proteomics and transcriptomics. The metabolic pathway analysis and multi-omics integration can consolidate the hypothesis of metabolic interactions and can even reveal unseen metabolic interactions, and consequently can be used to discover new biomarkers or therapy strategies. This integrative analysis can therefore reveal other metabolite sets leading to increased biomarkers performance, while promoting biomarkers acceptance by the community, by associating it with the mechanistic metabolic knowledge available.

## Figures and Tables

**Table 1:** Some examples of biomarkers, based on metabolomics of biofluids, to predict infection or/and the causative agent or/and disease severity or/and diseases outcome.

Biomarker Type, Models Predictability	Biofluid/Analytical Technique	Patients with a Defined Condition, Population Dimension	Ref.
Infection vs. non-infected and mechanical ventilated patients, Q^2^_(NMRS)_ = 0.789 & Q^2^_(GC-MS)_ = 0.971 Discriminate viral and bacterial pneumonia, Q^2^_(NMRS)_ = 0.789 & Q^2^_(GC-MS)_ = 0.971 90 days mortality, Q^2^_(NMRS)_ = 0.597 & Q^2^_(GC-MS)_ = 0.829	Plasma NMR & GC-MS	H1N1 viral pneumonia, n = 42 Non-infected patients, n = 31 With CAP, n = 30 Disease progression among patients with viral pneumonia, n = 21	[31]
Discriminate active from non-active tuberculosis, AUC = 0.914 or from CAP, AUC = 0.894	Plasma LC-MS	Active tuberculosis, n = 46 Non active tuberculosis infection, n = 30 With CAP, n = 30	[37]
Identify melioidosis, AUC = 1.0 Discriminate from bacteremia, AUC = 0.998	Plasma LC-MS	With *Burkholderia pseudomallei*, n = 22 Non-infected patients, n = 30 With bacteremia, n = 24	[38]
Sepsis, AUC = 0.719	Plasma GC-MS	Sepsis, n = 31 Healthy subjects, n = 23	[39]
Discriminate children with respiratory syncytial virus from healthy ones, Q^2^ = 0.76 Disease severity, Q^2^ = 0.81	Urine NMR	Children with respiratory syncytial virus, n = 55 Healthy children, n = 37	[40]
Infection in cancer patients with chemotherapy-associated neutropenia, AUC = 0.991	Plasma LC-MS	With infection, n = 14 Without infection, n = 25	[41]
Infection vs. healthy, Q^2^ = 0.820 Infection among other diseases states, Q^2^ = 0.828 Discriminate *S. pneumoniae* pneumonia from viral pneumonia, Q^2^ = 0.665 Discriminate from other bacterial pneumonia, Q^2^ = 0.680	Urine NMR	With *S. pneumococcal* pneumonia, n = 62 Healthy subjects, n = 115 Non-infectious metabolic stress, n = 55 With viral infection, n = 57 With pneumonia from other bacteria, n = 80	[42]
Sepsis (NMR), AUC = 0.94 Sepsis (LC-MS/MS), AUC = 0.97	Urine NMR & LC-MS/MS	Neonates with sepsis, n = 16 Healthy neonates, n = 16	[43]
Septic shock vs. healthy, AUC= 0.98 SIRS vs. healthy, AUC= 0.95 Septic shock vs. SIRS, AUC = 0.82	Serum NMR	Pediatric (neonates to 11 years old) Septic shock, n = 60 SIRS, n = 40 Healthy, n = 40	[44]
CAP severity, AUC = 0.911	Serum LC-MS/MS	Discovery cohort (n = 102); Validated cohort (n = 73)	[45]
Progression to ARDS, specificity = 1, sensitivity =1	Serum LC-MS/MS	With H1N1 virus infection, n = 25 that developed to ARDS, n = 17	[46]
Mortality, AUC = 0.75	Serum LC-MS/MS	*S. aureus* bacteremia, n = 200	[47]
90 days mortality, AUC = 0.91, sensitivity 0.82, specificity 0.91	Plasma DI-MS/MS	With CAP, n = 150	[23]
28 days mortality ROCI, AUC = 0.91 CAPSOD, AUC = 0.74	Plasma GC-MS& LC-MS	ROCI (patients with SIRS, sepsis, sepsis-induced ARDS), n = 90; CAPSOD, n = 149 (validation)	[48]
90 days mortality, AUC = 0.67	Plasma GC-MS& LC-MS	CAP patients that died, n = 15 that survived, n = 15	[49]
COVID-19 severity, AUC= 0.83 COVID-19 mortality, AUC = 0.760	Serum LC-MS/MS	COVID-19 patients, n = 120 for validation, n = 90	[50]
COVID-19 infection, AUC = 1.00 Mild vs. severe COVID-19, AUC = 0.708 Death from severe group, AUC = 0.737 Death from mild severe group, AUC = 0.865	Plasma LC-MS	Non infected, n = 10; Mild (n = 14) and severe (n = 11) COVID-19; Fatal COVID-19, n = 9	[51]
COVID-19 infection, specificity >0.96, sensitivity >0.83 Disease severity, specificity >0.80 and sensitivity >0.85	Plasma LC-MS/MS	With COVID-19, n = 442 Non-COVID-19, n = 350	[52]

Q^2^, score to predict a new sample based on a test data set.
